# AI-Powered Early Detection of Retinal Conditions: A Deep Learning Approach for Diabetic Retinopathy and Beyond

**DOI:** 10.1155/ijbi/6154285

**Published:** 2025-10-06

**Authors:** Ali Basim Mahdi, Zahraa A. Mousa Al-Ibraheemi, Zahraa Fadhil Kadhim, Raffef Jabar Abbrahim, Yaqeen Sameer Dhayool, Ghasaq Mankhey Jabbar, Sajjad A. Mohammed

**Affiliations:** Biomedical Engineering Department, Faculty of Engineering, University of Thi-Qar, Iraq

## Abstract

Various retinal conditions, such as diabetic macular edema (DME) and choroidal neovascularization (CNV), pose significant risks of visual impairment and vision loss. Early detection through automated and accurate and advanced systems can greatly enhance clinical outcomes for patients as well as for medical staff. This study is aimed at developing a deep learning–based model for the early detection of retinal diseases using OCT images. We utilized a publicly available retinal image dataset comprising images with DME, CNV, drusen, and normal cases. The Inception model was trained and validated using various evaluation metrics. Performance metrics, including accuracy, precision, recall, and *F*1 score, were calculated. The proposed model achieved an accuracy of 94.2%, with precision, recall, and *F*1 scores exceeding 92% across all classes. Statistical analysis demonstrated the robustness of the model across folds. Our findings highlight the potential of AI-powered systems in improving early detection of retinal conditions, paving the way for integration into clinical workflows. More efforts are needed to utilize it offline by making it available on ophthalmologist mobile devices to facilitate the diagnosis process and provide better service to patients.

## 1. Introduction

Diabetic retinopathy (DR) damages blood vessels in light-sensitive part of the eyes and results in blindness with a lack of discernible feeling, where initially, there are no symptoms [1]. Affordable technologies are required to support retinal damage early diagnosis.

Such diagnostic modalities, for example, fundus photography, fluorescein angiography, and optical coherence tomography (OCT) equipment, are pricey and only available in medical settings [2]. This challenge contributes to a significant obstacle in managing and controlling DR: the absence of a personalized risk model in addition to the inability to accurately predict the timing of the disease's onset and progression. So, creating such a model is so crucial and highly recommended.

On the other hand, with the manifestation of deep learning, in particular, convolutional neural networks (CNNs), a notable transformation happened. CNNs, owing to their ability to gain and extract hierarchical features from the data, have achieved important improvements in DR detection accuracy nowadays [3, 4]. Various digital technologies have initiated to present integrated solutions between improving diabetes management and providing DR screening; however, they still need more training to be used more easily by the medical experts [5–7]. Existing studies predominantly focus on individual retinal conditions like DR, with limited research on multicondition detection using a single AI model [8]. Moreover, many AI systems lack clinical applicability due to dataset biases or insufficient evaluation of robustness across folds. An example is the development of an application that ophthalmologists can download to their smartphones in order to precisely identify retinal disorders, which is regarded as a preliminary study [9].

In this study, we utilized a well-curated OCT dataset that includes retinal images categorized into four clinically significant classes: normal, choroidal neovascularization (CNV), diabetic macular edema (DME), and drusen. CNV and drusen are commonly associated with age-related macular degeneration (AMD), while DME is a key manifestation of diabetic retinal complications. The inclusion of these diverse pathologies enables the model to effectively distinguish between healthy and diseased retinas and to identify retinal abnormalities related to both diabetic and nondiabetic conditions. All images were carefully graded and verified by ophthalmologists and retinal specialists to ensure label accuracy.

This study bridges these gaps by developing a comprehensive deep learning model capable of detecting multiple retinal diseases using a robust methodology so that its primary goal was to develop a model that uses convolution neural networks to automatically and accurately identify different classes of retinal conditions.

Our contributions in this study are as follows:

1. We develop an end-to-end deep learning system with automatic feature extraction using CNNs.
2. Unlike other studies, where the CNN is trained from scratch, we use transfer learning to distill knowledge from pretrained networks, which helps in reducing training time while improving generalization for small datasets.
3. We assess the model using comprehensive performance metrics and highlight class-specific performance.

## 2. Methodology

### 2.1. Dataset Description

**Figure 1:**
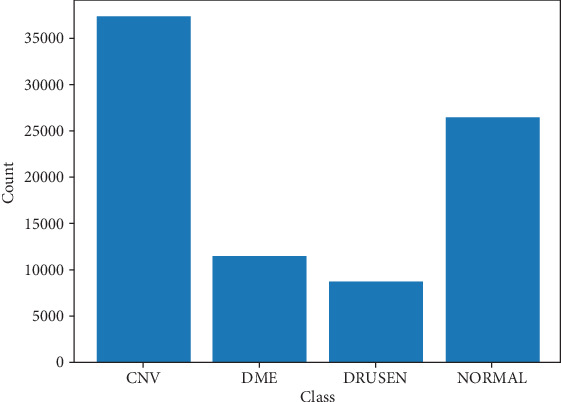
Class distribution.

**Figure 2:**
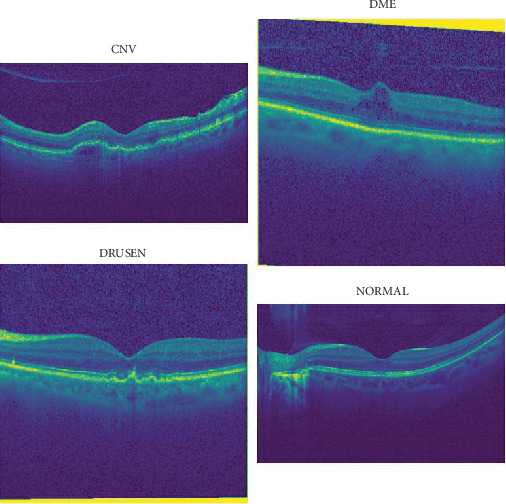
Image samples.

Images from OCT were chosen from adult patient retrospective cohorts across multiple institutions. The dataset is arranged into three folders (train, test, and validation), each of which has subfolders for the following image classes: normal, CNV, DME, and drusen. Images are categorized into four directories: CNV, DME, drusen, and normal. Images are categorized into four directories: CNV, DME, drusen, and normal, labeled as (disease)-(randomized patient ID). Prior to training, every image was subjected to a three-tiered grading system for label verification and correction. This involved undergraduate and medical students performing initial quality control, ophthalmologists independently rating the images, and senior retinal specialists verifying the labels last. Two ophthalmologists independently rated a validation subset of 993 images; in this research, we use this subset for testing our model performance [10].  Table 1 provides a detailed overview of OCT image categories, including the counts for each class. Figures 1 and 2 present sample OCT images alongside a column chart illustrating the distribution of image counts across the various categories.

### 2.2. Data Preprocessing

The images underwent a preprocessing stage to improve their quality. The size of the images collected from Kaggle was changed to vary the size and resolution of the images to 299 × 299 pixels using the Inception model. The different CNNs were classified on the training data to perform the classification [11, 12].

#### 2.2.1. Data Resizing

**Figure 3:**
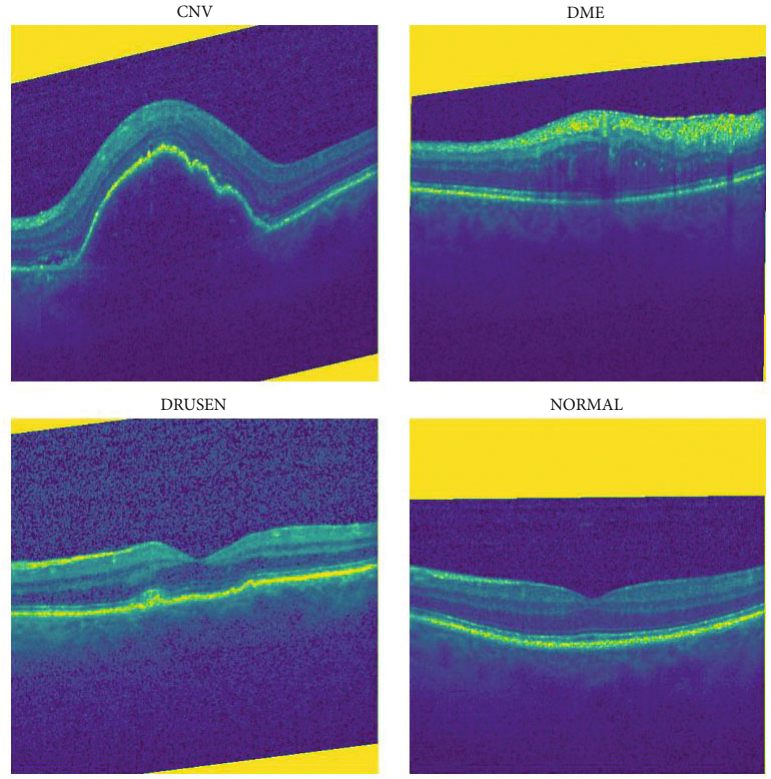
Resized images.

The standardization process ensures that all images with a size of 299 × 299 pixels are processed effectively during training and testing. Resized images are shown in Figure 3. In addition, OCT images are normalized to the range of [0,1].

#### 2.2.2. Data Augmentation

Due to the imbalance in the dataset, training a deep learning model on such a distribution will lead to biased results for the dominant class of data. For this, we use data augmentation techniques to create a more balanced dataset in the training process [13]. However, due to the sensitive nature, only the following transforms are used: shear transformation, random zoom, and random clipping. This helps the model become more robust to new images while training and testing as well as balancing the dataset.

#### 2.2.3. Data Split

In machine learning of retinal OCT images, we divide the data into training, validation, and testing as 70%, 20%, and 10%, respectively. The validation set is used to validate the model's performance during training, and the testing set is used for the final evaluation of the model after the training is complete.

### 2.3. Inception and VGG16

**Figure 4:**
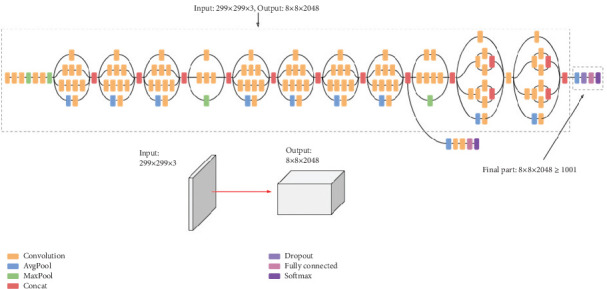
Inception architecture.

The Inception model, as shown in Figure 4, was selected for its ability to efficiently extract multiscale features, which are essential for the variable textures and patterns seen in retinal images. Unlike ResNet or DenseNet, Inception's architecture optimizes computational efficiency while maintaining high accuracy. It is the latest architecture used in image classification and detection by optimizing the computational resources within the network. It is used to efficiently capture various features at different levels. It allows deep networks with increased width and depth while managing computational complexity. It also enhances speed and performance in image processing tasks [14]. Initially, the training is done using the VGG16 architecture, with no additional layers or modifications, except for the last layer (the decision layer). And it was also compared with Inception (also without any additional layers or modification). This helped us to choose between the two architectures for our proposed network.

### 2.4. Proposed Network

We use the transfer learning method to take advantage of the pretrained model's knowledge—in this case, Inception on 1000 classes of the ImageNet dataset—to improve and speed up the performance on a new and often smaller dataset. This method is often used by freezing all layers except the last layer, but we freeze all the layers except the last four, which produced a higher performance and allowed the model to be adjusted to our data while retaining the important traits that were learnt during the first training. This method lessens the chance of overfitting while also cutting down on the amount of computing power and time needed to train the model from the start [15].

Next, we add a fully connected layer with 256 and 128 neurons, respectively, to augment the Inception architecture so that the model can learn intricate representations for the classification problem through these extra layers [16]. We employed categorical cross-entropy as our loss function during training; the reason is because the aim of a multiclass classification problem is to assign an input to one of multiple possible categories; this loss function works well because it measures the difference between the genuine distribution, which is represented by the ground truth labels, and the expected probability distribution produced by the model, and we can express that categorical cross-entropy is mathematically as follows:

(1) $$\begin{array}{c} \text{Loss}=-\overset{C}{\underset{i=1}{\sum }}\;y_{i}\log\left({\overset{ˆ}{y}}_{i}\right), \end{array}$$

where *C* is the number of classes, *y*_*i*_ is the true label (1 if the class is correct, 0 otherwise), and ${\overset{ˆ}{y}}_{i}$ is the predicted probability for class *i*. In order to encourage the model to produce predictions that are as near to the actual labels as feasible, the loss function penalizes the model more when the predicted probability is distant from the true label [17].

We added a layer of dropout regularization of 0.5 after the fully connected layers, just before the softmax activation function. During training, dropout is used to arbitrarily “drop out,” or eliminate a portion of the neurons, forcing the network to produce a more robust set of features and avoiding the model from depending too much on any one neuron. This regularization keeps the model's applicability to the test data intact [18].

The softmax function, applied to the output layer, converts the model's raw output scores into probabilities, ensuring that the sum of all probabilities equals one. The softmax function is mathematically defined as follows:

(2) $$\begin{array}{c} \sigma {\left(z\right)}_{j}=\frac{e^{z_{j}}}{\sum_{k=1}^{C}\;e^{z_{k}}}, \end{array}$$

where *z*_*j*_ represents the raw output score for class *j* and *C* is the total number of classes. The softmax function ensures that the network's predictions are interpretable as probabilities, making it easier to assign an input to a specific class based on the highest probability.

**Figure 5:**
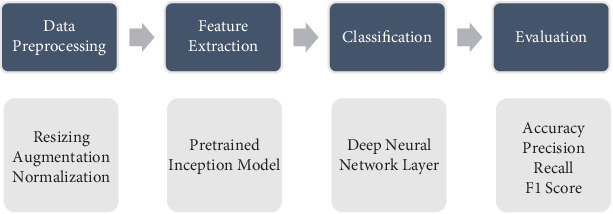
A flow diagram of the proposed method.

All the training is conducted using the Adam optimizer, a popular optimization algorithm that combines the benefits of the AdaGrad and RMSProp algorithms. Adam modifies the learning rate according to estimates of the gradients' first and second moments for each parameter. This leads to better performance and faster convergence [19]. To help the model generalize better for the minority classes during training and testing, we use class weights, which help adjust the model's loss function by assigning a higher weight to minority classes and a lower weight to the majority class. With this adjustment, we were able to use the pretrained Inception model's learned features to tailor it to our dataset and our classification objective. The diagram in Figure 5 shows our proposed method.

### 2.5. Evaluation Metrics

When evaluating a deep learning model for retinopathy classification, accuracy, precision, recall, and *F*1 score are important metrics that help us to understand how well the model performs, and these metrics are expressed as follows:

(3) $$\begin{array}{c} \text{Accuracy}=\frac{\text{TP}+\text{TN}}{\text{TP}+\text{TN}+\text{FP}+\text{FN}}, \end{array}$$

(4) $$\begin{array}{c} \text{Precision}=\frac{\text{TP}}{\text{TP}+\text{FP}}, \end{array}$$

(5) $$\begin{array}{c} \text{Recall}=\frac{\text{TP}}{\text{TP}+\text{FN}}, \end{array}$$

(6) $$\begin{array}{c} F1\text{ score}=2\cdot \frac{\text{precision}\cdot \text{recall}}{\text{precision}+\text{recall}}, \end{array}$$

where true positive is TP, true negative is TN, false positive is FP, and false negative is FN. The accuracy measures the percentage of correctly classified cases in the total, but we should not rely solely on it; this is why we use precision to measure how well the model detects actual cases of retinopathy without producing an excessive number of FPs. Conversely, recall assesses the capacity of the model to identify every class of retinopathy that really occurs to make sure that no cases are overlooked. Lastly, to take into account both FPs and FNs, the *F*1 score provides a balanced assessment of the model's performance by combining precision and recall into a single measure [20].

**Figure 6:**
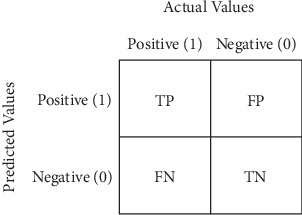
Confusion matrix grid.

In addition, we use a confusion matrix, as in Figure 6, which is a performance evaluation tool used in machine learning, particularly in classification. It summarizes the performance of a classification model by tabulating the predicted classes against the actual classes [21]. In the context of retinopathy multiclass classification, it would look something like this.

## 3. Results and Discussion

In this section, we present the results obtained from using transfer learning with the Inception architecture for retinopathy classification using OCT images. We evaluate the performance of our method using the metrics discussed in the previous section, such as accuracy, precision, recall, and *F*1 score, the confusion matrix, and classification reports. A detailed analysis of the experimental findings is conducted, emphasizing the strengths and weaknesses of the proposed model through systematic comparisons with existing methods.

The accuracy evaluation serves as a standard by which to compare different classification techniques. The model's initial accuracy on the training dataset is around 80.43%, which indicates that 80.43% of the training samples are properly identified. Training accuracy rises with each epoch, suggesting that the model is effectively learning from the input. Validation accuracy begins marginally higher than training accuracy and increases through the epochs, reaching around 91.28% by the fifth epoch. Validation accuracy evaluates performance on a different dataset of 16,696 images that are not used during training. This implies a good generalization to unknown data (for the Inception network, refer to Table 2 for loss and accuracy values over five epochs).

In contrast, the initial VGG16 model, trained without unfreezing and with no additional custom layers, exhibited different results, as depicted in Table 3. These results show that the performance of the Inception architecture for this data is better than VGG16, which is why Inception was selected as a pretrained model for our proposed network.

**Figure 7:**
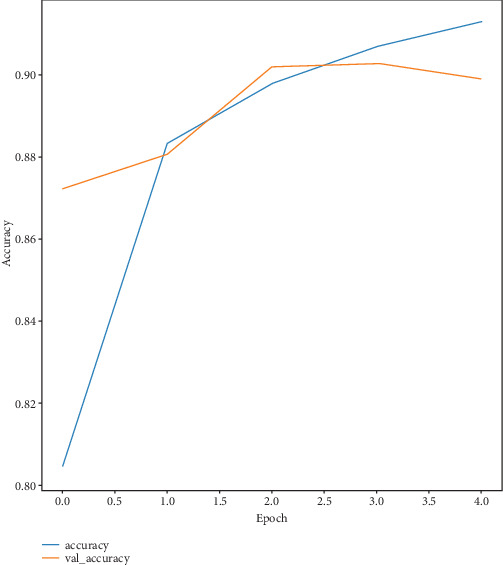
Training and validation accuracy curves for Inception per epoch.

**Figure 8:**
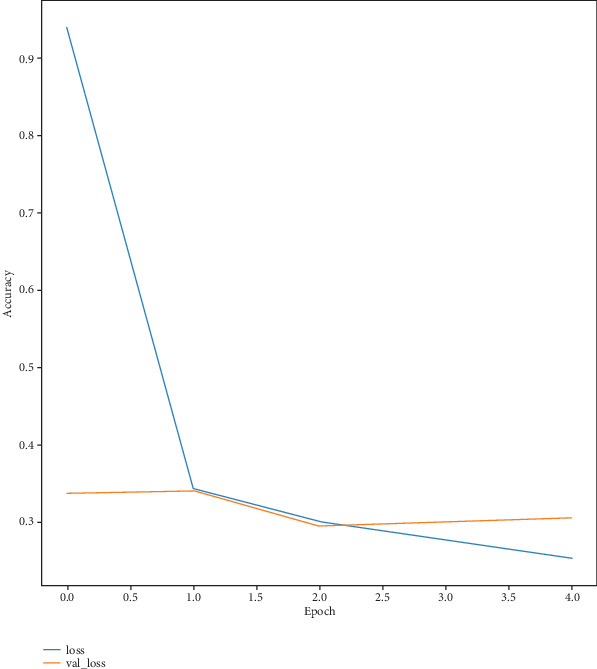
Training and validation loss for Inception per epoch.

**Figure 9:**
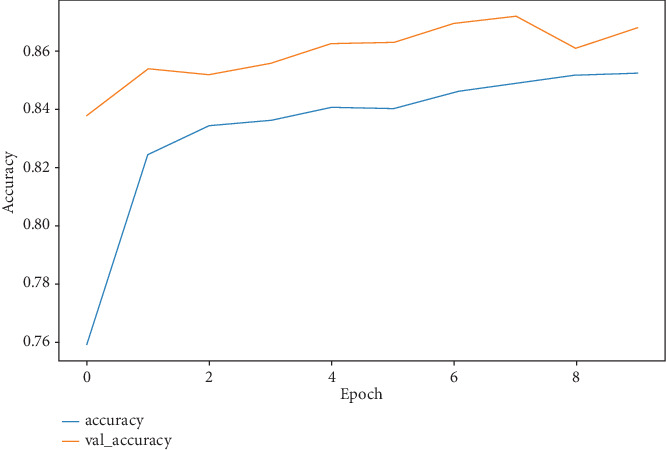
Training and validation accuracy of VGG16 for multiple epochs.

The model has a notable training loss of 0.44 at first, which indicates the difference between expected and actual values, but this loss diminishes across epochs, suggesting that predictions are getting more accurate. The validation loss shows how well the model generalizes to new data; it also reduces over epochs. The relationship between decreasing loss and increasing accuracy highlights how well the model learns from and adapts to new data. As the loss decreases, the model's accuracy rises on the training and validation datasets. This pattern points to enhanced output and rapid learning.  Figure 7 shows the training and validation accuracy per epoch of the Inception network, and Figure 8 shows the training and validation loss over epochs. Also, Figure 9 shows clearly the training and validation accuracy of VGG16 for multiple epochs.

A total of 968 OCT images graded by two ophthalmologists were used to evaluate this model against a ground truth. The classification results of the model were generally correct and had an accuracy of 90.73%. The model presented a very low FP probability which means that it predicted positives mostly very reliably with a precision of 91.36%. The model also has a recall of 90.73%, meaning it will be able to find real positive cases with high accuracy, but less missed detections. The model's ability to effectively balance recall and precision is demonstrated by its *F*1 score of 90.78%, which shows the performance in correctly identifying genuine positives while preventing FPs. Collectively, these metrics offer a comprehensive evaluation of the model's effectiveness as presented in Table 4. To compare our results to the most recent studies on retinopathy classification and detection, we used VGG16 for classification and found an accuracy of 78.3%. Meanwhile, we have used Inception and obtained 90.73% [22]. This team used the fast AI library, and they found an accuracy of 80%. However, we used Inception and obtained low loss and high accuracy of approximately 90.73%. This team used new method examination results indicating that more than minimal DR cases were detected with an AUC of 0.839 by the EyeCheck algorithm and an AUC of 0.821 for the Challenge2009 algorithm, a statistically nonsignificant difference. If either of the algorithms detected DR in combination, the AUC for detection was 0.86, but we used VGG16 and obtained an accuracy of 86% and then used Inception and obtained an accuracy of 90.73%. Shekhar and Thakur [23] used CNN and found an accuracy of 80%. Meanwhile, we used Inception and obtained 90.73%.

The model's performance assessment across the four classes—CNV, DME, drusen, and normal—can be found in the classification report in Table 5 to provide a clearer understanding of how well each class is classified relative to others, with 242 cases for each class and a total of 968 support.

**Figure 10:**
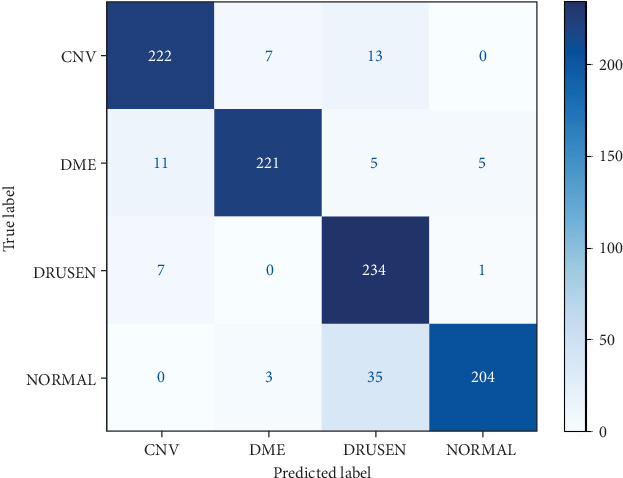
Confusion matrix results for pretrained Inception.

Also, the model's performance in each class is shown in the confusion matrix output in Figure 10. The count of TP, TN, FP, and FN forecasts is displayed on a grid. With the help of this matrix, we can examine the model's potential overall performance, which makes it simpler to identify any imbalances in prediction performance and identify which particular regions may require fine-tuning, as well as the specific weaknesses and strengths of the model.

Although the current model is focused on multiclass classification of retinal pathologies using OCT images, it provides a foundational step toward developing more personalized and predictive tools in ophthalmology. By distinguishing between conditions such as DME, CNV, drusen, and normal retina with high precision, the model helps to stratify patients based on the specific type and stage of disease. This stratification could be extended in future work by integrating longitudinal OCT data or clinical history, enabling the prediction of disease progression over time. As such, while the model does not currently predict the exact timing of disease onset or progression, it creates the structural basis necessary for such personalized modeling in future studies.

## 4. Conclusion

Nowadays, retinopathy is continuously becoming a thorny problem among adults, which needs a huge demand for resolving the issue in the early stages to eliminate the chances of blindness. Currently, Al and ML are employed as assessment methods to diagnose and classify multiple retinopathy diseases. This article provides the findings from our investigation on machine learning–based retinopathy diagnosis. Inception enhances retinopathy diagnosis, improving patient outcomes through accurate, sensitive fundus image analysis and early detection. The goal is to create a reliable model for OCT image-based retinopathy detection and diagnosis using machine learning and Inception architecture. In the coming days, this work can be considered a foundation for more models that employ a larger dataset to identify different stages of DR with higher accuracy and fewer efforts. While the current study focuses on multiclass classification of retinal pathologies using OCT images, future research may extend this model by incorporating patient-specific clinical data and longitudinal OCT scans. This would enable more personalized risk assessments and potentially allow for accurate prediction of disease onset and progression, aligning with the goal of developing intelligent systems for proactive ophthalmic care.

## Figures and Tables

**Table 1:** Dataset class counts.

**Class**	**Count**
CNV	37,205
DME	11,348
Drusen	8616
Normal	26,315

**Table 2:** Loss and accuracy values of the proposed Inception network for training and validation across five epochs.

**Epoch**	**Loss**	**Accuracy**	**Validation loss**	**Validation accuracy**
1	0.9403	80.44%	0.3362	87.21%
2	0.3426	88.33%	0.3391	88.05%
3	0.2994	89.76%	0.2936	90.19%
4	0.2732	90.68%	0.2999	90.27%
5	0.2517	91.28%	0.3044	89.90%

**Table 3:** Results of pretrained VGG16 using only transfer learning with no unfreezing.

**Epoch**	**Training loss**	**Training accuracy**	**Validation loss**	**Validation accuracy**
1	0.489468	82.45%	0.415917	85.39%
2	0.462101	83.43%	0.404933	85.18%
3	0.456036	83.62%	0.392021	85.57%
4	0.448369	84.06%	0.381004	86.25%
5	0.4233	84.90%	0.3658	87.20%

**Table 4:** Accuracy, precision, recall, and *F*1 score metrics for testing data.

**Accuracy**	**Precision**	**Recall**	**F**1**score**
90.2%	91.36%	90.73%	90.78%

**Table 5:** Detailed classification report for each class.

**Class**	**Precision**	**Recall**	**F**1**score**	**Support**
CNV	0.85	0.92	0.88	242
DME	0.93	0.87	0.90	242
Drusen	0.89	0.81	0.85	242
Normal	0.92	0.99	0.96	242
Average	0.90	0.90	0.90	968

## Data Availability

The data used in this research is from an open-source dataset that is publicly available here: https://www.kaggle.com/datasets/paultimothymooney/kermany2018.
